# Light Manipulation in Inhomogeneous Liquid Flow and Its Application in Biochemical Sensing

**DOI:** 10.3390/mi9040163

**Published:** 2018-04-02

**Authors:** Yunfeng Zuo, Xiaoqiang Zhu, Yang Shi, Li Liang, Yi Yang

**Affiliations:** School of Physics and Technology, Wuhan University, Wuhan 430070, China; zuoyf@whu.edu.cn (Y.Z.); daveria@whu.edu.cn (X.Z.); shiyang@whu.edu.cn (Y.S.); lianglill@whu.edu.cn (L.L.)

**Keywords:** optofluidics, inhomogeneous medium, light manipulation, biochemical sensing

## Abstract

Light manipulation has always been the fundamental subject in the field of optics since centuries ago. Traditional optical devices are usually designed using glasses and other materials, such as semiconductors and metals. Optofluidics is the combination of microfluidics and optics, which brings a host of new advantages to conventional solid systems. The capabilities of light manipulation and biochemical sensing are inherent alongside the emergence of optofluidics. This new research area promotes advancements in optics, biology, and chemistry. The development of fast, accurate, low-cost, and small-sized biochemical micro-sensors is an urgent demand for real-time monitoring. However, the fluid flow in the on-chip sensor is usually non-uniformed, which is a new and emerging challenge for the accuracy of optical detection. It is significant to reveal the principle of light propagation in an inhomogeneous liquid flow and the interaction between biochemical samples and light in flowing liquids. In this review, we summarize the current state of optofluidic lab-on-a-chip techniques from the perspective of light modulation by the unique dynamic properties of fluid in heterogeneous media, such as diffusion, heat transfer, and centrifugation etc. Furthermore, this review introduces several novel photonic phenomena in an inhomogeneous liquid flow and demonstrates their application in biochemical sensing.

## 1. Introduction

The term ’optofluidics’ appeared in 2003 at the California Institute of Technology in Pasadena, owing to the development of microfluidics [1]. Microfluidics is a promising field aimed at fluidic manipulation with various applications in fields such as biochemistry technology, molecular analysis, chemical synthesis, and energy conversion [2,3,4,5,6,7], and especially in field of single-cell biology [8,9,10]. Pagliara et al., developed a microfluidic assay to confine single cells. This design provided an efficient tool for single cell detection [11]. Optofluidics is a new analytical field that focuses on the integration of optics and microfluidics, providing many unique characteristics for enhancing the performance and simplifying the design of micro-electromechanical systems [12,13,14]. Over the past decades, this new field has rapidly developed and has been applied in many areas, such as biosensors, biomedical analyses, energy production, optical imaging and many other optical systems [15,16,17,18,19,20,21,22]. The manipulation of light, such as in light routing, focusing, bending etc. plays an important role in lab-on-a-chip optofluidic systems. Recent advancement in optofluidics has demonstrated a new class of microsystems that exploits microfluidic flow to manipulate light in the microchannel, realizing various optical devices and functionalities [23] such as liquid microlenses [24,25], prisms [26,27], gratings [28,29], switch [30,31], and dye lasers [32,33].

For the purpose of light manipulation, optofluidic lab-on-a-chip techniques have several novel merits that cannot be found in conventional solid-based optical systems [1,12,13]. Optofluidics makes full use of liquids to manipulate light beams. Liquids are natural optical materials that possess greater tunability in their refractive index and a greater flexibility in shape than their solid equivalents. Pure liquid optofluidic devices fabricated by polydimethylsiloxane (PDMS) have several useful characteristics. First, the optical property of the fluid media and refractive index (RI) distribution can be easily changed by replacing one fluid with another or changing the flow rates. Second, the interface between the two fluids can be optically smooth and that can reduce the propagation loss of the light beams. Finally, there is the ability to create a gradient refractive index (GRIN) through the process of liquids diffusion. The optical properties, such as refractive index, absorption, and fluorescence etc., and the physical properties, such as the magnetic susceptibility and electrical conductivity of the optofluidic devices can be changed easily, dynamically, and continuously. These properties can be used to design novel devices.

As mentioned above, optofluidic devices and systems can be tuned by changing the refractive index distribution of its liquid. Conventional light manipulation techniques change the refractive index by introducing an external electrical field, magnetic field, temperature field, acoustic field, or mechanical strain. Optofluidic approaches such as pumping and mixing can be used for changing the liquids or its composition. If the liquid is a solution, by integrating a concentration generator and mixer in the device, the composition and concentration can be adjusted readily (e.g., the refractive index of CaCl_2_ solution can be changed from 1.334 to 1.445). In principle, any liquids that are compatible with the microchannel and process excellent light stability can be used to form the optofluidic devices. The refractive index of the common liquids ranges from 1.300 (2,2,2-Trifluoroethanol) to 1.749 (methylene iodide). Since the flow streams of the optofluidic device can be replaced by pumping different liquids, a large relative refractive index change *σ* (∆*n*/*n*) can be achieved. Where *σ* is defined as the ratio between the RI difference and the RI of the initial liquid (e.g., if replacing deionized water by Benzothiazole, *σ* = 0.23). In an extreme case, replacing air by a liquid, a relative high *σ* ~1 can be achieved, while other light manipulation techniques suffer from a small value of σ owing to the fixed optical material. Table 1 demonstrates the performance of the different light manipulation techniques [1]. 

In this review, we provide an overview of optofluidic lab-on-a-chip techniques based on an inhomogeneous liquid flow for light manipulation. First, we describe the fundamental concepts and principles associated with liquids and light manipulation in optofluidic systems. Then, previous optofluidic lab-on-a-chip techniques will be categorized in two kinds from the perspective of manipulation of liquid-liquid interface and manipulation of the liquid gradient refractive index distribution. In pure liquid optofluidic systems, the geometric interface between liquids and the refractive index distribution of liquids dominate the light manipulation. Finally, we introduce several novel photonic phenomena due to the interaction of fluids and light in micro- or nano-scale and demonstrate their application in micro-/nano-optical devices and biological/chemical microsensors, etc. We then discuss the outlook, research trends in light manipulation in an inhomogeneous liquid flow and its potential applications in new and ground-breaking research areas.

## 2. Fundamental Concepts and Principles

As stated above, optofluidics is the combination of microfluidics and optics. The fundamental goal of light manipulation in optofluidics is to form a proper refractive index profile through manipulating the fluid dynamics processes in a microchannel. For the purpose of light manipulation in an inhomogeneous liquid flow, precise control of the liquid is of vital importance. The physical phenomena that exist in microfluidic systems also occur in optofluidic systems. To reveal the fluid dynamics processes in optofluidic devices and differentiate primary from secondary, several dimensionless numbers are used for expressing the ratio of the fluid dynamics phenomena [34]. 

### 2.1. The Reynolds Number

To research the flow streams in a microchannel, the Reynolds number (*Re*) is frequently involved to characterize different flow types. It is one of the most fundamental concepts in microfluidics.

(1) Re=ρVL/μ

where *ρ* is the fluid density, *V* is the average flow velocity, *L* is the channel hydraulic diameter and *μ* is the kinematic viscosity of the fluid. Hydraulic diameter is defined as *L* = 4*A/P*, where *A* is the cross-sectional area of the channel, *P* is the wetted perimeter of the cross-section. When *Re* < 2300, the state of the flow is laminar and is generally smooth and predictable. As *Re* increases, the first features of inertia become apparent. When *Re* > 4000, the flow becomes an unpredictable, irregular turbulent flow [35]. At the microchannel scale, *Re* is so small that the flow is laminar, which provides a stable fluidic condition for optical waveguide. 

### 2.2. The Dean Number

In a condition of relative high *Re*, the inertia role of the fluids cannot be ignored. When the fluid is moving through a curved microchannel, whose radius of curvature *R* is comparatively much larger than the hydraulic diameter of the microchannel *L*, centrifugal forces on the liquid flow will give rise to a secondary motion. In the situation of the secondary Dean flow, the fluid in the center of the microchannel will be driven to the outer side of the cured microchannel. While the fluid close to the channel wall will be swept towards the inside [36]. The Dean flow can be described and defined by a dimensionless Dean number (*De*), which can be expressed as:

(2) De=δ12Re

where δ *= L/R* is a geometrical parameter. When the liquids are designate, the feature size of the curved microchannel and the *Pe* of the liquid streams determine the Dean number and the liquid transverse movement. *De* reflects the relative relation between centrifugal forces and viscous forces.

### 2.3. The Convective–Diffusive Transport

The inhomogeneous liquid flows in optofluidic devices are prominently governed by the diffusion and convection process. And the convection–diffusion equation is applied to describe the convective–diffusive transport [37]. This equation is a combination of the diffusion and convection equations, and describes the transfer of molecules, energy, or other physical quantities inside a physical system.

The general equation can be formulated as:

(3) $$\frac{\partial C}{\partial t}=D\nabla^{2}C-U\nabla C+S$$

where C is the concentration of the liquid, *t* is time, *D* is the diffusion coefficient of the solute, and U represents the average velocity in the microchannel. *S* describes the contribution of the chemical reaction. In almost every situation of optofluidics, there is no chemical reaction between liquids, and it has *R* = 0. The first term on the right side represents the diffusion process while the second term corresponds to the convection process. For a steady-state and passive flow (*S* = 0), the concentration does not change with time, thus the term *∂C/∂t* leads to zero. In most optofluidic devices, the control of convective–diffusive transport is the fundamental principle and the main method of light manipulation. The convection process plays an important role in optofluidic devices based on the manipulation of the liquid-liquid interface. While in the gradient refractive index (GRIN) and the optofluidic transformation optical devices, both of which are based on manipulation of the gradient refractive index, the diffusion process is dominant.

### 2.4. The Péclet Number

The relative importance of convection to diffusion is characterized by the dimensionless Péclet number:

(4) Pe=UW/D

where U is the average velocity, *W* is the width of the microchannel and *D* is the diffusion coefficient. The competition between convection and diffusion, embodied in the Péclet number, forms the basis of a number of optofluidic techniques for light manipulation. 

## 3. Manipulation of the Liquid-Liquid Interface

A great number of optofluidic devices rely on the interface formed between streams of flowing liquids in a microfluidic channel. The inhomogeneous liquid flow and the step-refractive index distribution are formed by manipulating the liquid-liquid interface through various approaches such as hydrodynamic focusing, Dean flow, and two-phase flow etc. [38].

### 3.1. Hydrodynamic Focusing

In order to achieve precise control of the liquid-core/liquid-cladding system, the concept of hydrodynamic focusing is introduced. Various optofluidic devices are designed based on hydrodynamic focusing, such as step-refractive index waveguides, prisms, lens, and optical switch etc. A typical hydrodynamic focusing model is generally made of three flow streams: a core flow and two cladding flows [39]. In a simplified model, the micro-channel and velocity profile are symmetrical. The two laminar sheath flows with equal flow rates focus the core flow stream, the width of which can be adjusted from a few micrometers to hundreds of micrometers. Under a low Reynolds number the ratio between the width of the focused (*W_f_*) flow and that of the main channel (*W_m_*) is expressed as:

(5) $$\frac{W_{f}}{W_{m}}=\frac{Q_{i}}{\alpha \left(Q_{i}+Q_{c1}+Q_{c2}\right)}$$

where $Q_{i}$ and $Q_{c}$($Q_{c1}$, $Q_{c2}$) are the flow rates of the core and the sheath flow streams, respectively. *α* represents the velocity ratio $\alpha ={\bar{v}}_{f}/{\bar{v}}_{o}$. The equation reveals that the widths of the focused streams depend on the ratio of *Q_core_* and *Q_clad_* in the microfluidic channel under the effect of hydrodynamic force. By taking the convection–diffusion transport equation into account and applying it in the hydrodynamic focusing model, the normalized concentration distribution for the core flow and the cladding flows under dynamic equilibrium-state in the microchannel can be expressed as [40]:

(6) $$C_{core}^{*}\left(x^{*},y^{*}\right)=r+\frac{2}{\pi }\overset{\infty }{\underset{n=1}{\sum }}\frac{\sin\left(nr\pi \right)}{n}\cos\left[y^{*}n\pi \right]\;\times \exp\left[\frac{1}{2}x^{*}\left(Pe-x^{*}\sqrt{Pe^{2}+4n^{2}\pi^{2}}\right)\right]$$

(7) $$C_{clad}^{*}\left(x^{*},y^{*}\right)=1-r+\frac{2}{\pi }\overset{\infty }{\underset{n=1}{\sum }}\frac{\sin\left[n\left(1-r\right)\pi \right]}{n}\cos\left[(1-y^{*}\right)n\pi ]\;\times \exp\left[\frac{1}{2}x^{*}\left(Pe-x^{*}\sqrt{Pe^{2}+4n^{2}\pi^{2}}\right)\right]$$

where *Pe* is the Péclet number and *r* is the initial interface boundary ratio between the core and cladding fluids *r =* 1/(1 + 2*βκ*) (*β* = *μ*_1_/*μ*_2_), the dynamic viscosity ratio between the cladding and the core flows, and *κ* = $Q_{2}/Q_{1}$, the ratio of the flow rates of the cladding and the core streams). As the concentration of the solute is demonstrated, the index profile n(x*, y*) can be formulated as:

(8) $$n\left(x^{*},y^{*}\right)=\left[C_{clad}^{*}\left(x^{*},y^{*}\right)\times n_{clad}\right]+\left[C_{core}^{*}\left(x^{*},y^{*}\right)\times n_{core}\right]$$

**Figure 1:**
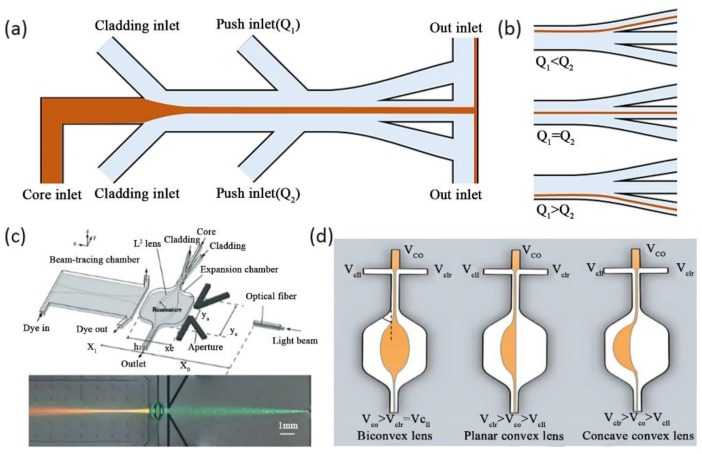
(**a**) Schematic of the liquid-core/liquid-cladding waveguide; (**b**) Verification of light output switching by changing the flow rates; (**c**) Schematic of a pure liquid lens formed by hydrodynamic focusing of three flow streams in a specially designed chip; (**d**) Schematics of the formations of the tunable liquid microlenses. Images reproduced with permission from [43,44].

In a condition of relative high *Pe*, the convection process dominates the fluid transportation in the microchannel. The RI distribution appears in a state of step-index profile. By changing the flow rates of the three independent flow streams, the width of the focused core flow can be adjusted, ranging from hundreds of micrometers to a few micrometers, even down to 50 nanometers [41]. The position of the focused core flow can be adjusted by introducing cladding flows with different flow rates. Based on hydrodynamic focusing, various optical devices and elements have been realized, such as waveguides and lenses [42,43,44,45]. As shown in Figure 1a Wolfe et al., demonstrated a design of pure liquid optical waveguides [42]. These waveguides were constructed by using a three flow streams system, which is a typical hydrodynamic focusing module. An aqueous solution 5M CaCl_2_ (*n* = 1.445) and deionizer water (*n* = 1.333) were introduced as the high RI core flow and the low RI cladding flows, respectively. Through adjusting the fluid flow rate, the width and the position of the core flow can be reconfigured to allow the flexible manipulation of light dynamically and continuously in real-time. Manipulating the rate of flow or changing the ingredient of the liquids will modulate the optical and physical properties of the optofluidic systems. 

By changing the flow rates of the cladding flows, the path of the core flow and then that of the light can be determined. An extra set of inlets were added to this liquid waveguide downstream of the initial inlets. These extra inlets were independently controlled to switch the core flow from one output to another without deforming the core flow at the junction point. Thus, this waveguide will maintain a low level of optical loss during output switching. The three switch states were shown in Figure 1b. The output light was controlled easily by changing the flow rates of the “push” inlets. The responding time of this pure liquid switchable waveguide was about 2 s.

As shown in Figure 1c, Tang et al., first demonstrated a pure liquid lens formed by the hydrodynamic focusing of three flow streams in a specially designed chip [43]. The liquid lens was formed in an expanded chamber after the hydrodynamic focusing occurred. The liquid-liquid interface of the core flow and the cladding flows fill the shape of the expanded chamber. In order to maintain the stability and the ideal lens shape, the length-to-width ratio of the expanded chamber was chosen to be 1:1. These lenses process high tunability as different lens shape and the curvature radius can be realized through adjusting the three flows. In the condition of a fixed total flow rate and equal flow rates of both cladding flow liquids, the curvature radius of the convex lens increased alongside an increase in the flow rate of the core flow. Thus, a wide range of focal distance of the liquid lens from ~12 mm to ~6 mm can be obtained through the variation of the rate of the core flow. Seow et al., also reported a similar design for light collimation and focusing [44]. Three types of liquid lens have been demonstrated by tuning these three flow streams as shown in Figure 1d. CaCl_2_ solution was used as the high *RI* core liquid (*RI* = 1.46) and the flow rate was *V_core_*. Isopropanol solution (*RI* = 1.33) was used as the low *RI* cladding liquid with flow rates of *V_c_*_l1_ and *V_c_*_l2_, respectively. If the flow rates of these two cladding flow streams are the same (*V_core_* > *V_c_*_l1_ = *V_c_*_l2_), a liquid biconvex lens will be formed in the expanded chamber. When *V_c_*_l2_ was increased and higher than *V_core_*, the curvature radius of the right interface increased at the same time. A planar convex lens will be formed when the curvature radius reaches infinity. By further increasing the flow rates of the right inlet *V_c_*_l2_, it will be reconfigured as a concave convex lens.

Although 2D hydrodynamic focusing is suitable for many applications, these 2D optofluidic devices suffer from large optical loss in vertical direction. The liquid distribution of the 2D devices can only be tuned in the horizontal direction. Considering a microchannel with a width of 200 µm and a height of 100 µm, the width of the core flow can be adjusted from ~200 µm to a few micrometers, however, the height of the core remains 100 µm. The interaction between the microchannel and the core flow limits the performance and tunability of the optofluidic systems. Recent advancements in the fabrication of complex microchannels make it possible to fabricate 3D optofludic devices. A curved liquid channel can also be used for forming 3D liquid systems through the effect of Dean flow.

### 3.2. Dean Flow

**Figure 2:**
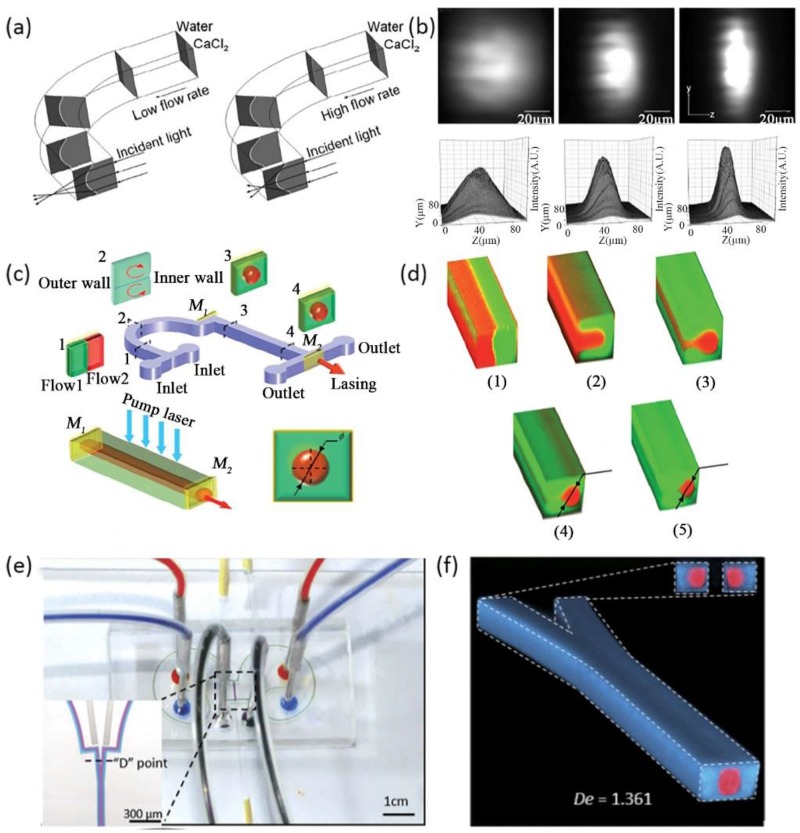
(**a**,**b**) The pure liquid optofluidic lens. The mechanism of the hydrodynamically tunable optofluidic cylindrical microlens through Dean flow; (**c**,**d**) The schematic illustration of the 3D liquid waveguide dye laser; (**e**,**f**) Switchable 3D optofluidic Y-branch waveguides tuned by Dean flows. Images reproduced with permission from [47,49,51].

The phenomenon of Dean flow is a novel tool for researchers, which makes it possible to control liquid in the vertical direction. Various novel optofluidic devices have been reported based on Dean flow, such as 3D drifting waveguides [46,47], cylindrical microlens in the *Z*-axis [48], 3D dye laser [49], and 3D lens [50] etc. Compared with traditional 3D structures dependent on the intricate fabrication and design of microchannels, optofluidic devices based on Dean flow poses the advantages of easy fabrication and high tunablility. Taking the advantages of Dean flow, Mao et al. demonstrated a cylindrical microlens [47]. The liquid cylindrical microlens was generated by the interface between a 5 M CaCl_2_ solution (*RI* = 1.445) and deionized water (*RI* = 1.333). A curved liquid interface was formed through the centrifugal force generated by the Dean flow. When the two liquid flows moved through the 90 degree curved microchannel, the inner flow of the CaCl_2_ solution bowed into the DI water and a secondary motion arose in the cross-section of the microchannel. The curved interface between the two liquids formed a planar convex cylindrical lens, as shown in Figure 2a. By changing the flow rates of each flow stream, the geometry of the liquid-liquid interface can be easily modulated, which means that the optical properties of the liquid lens are governed by the flow rate. Figure 2b shows the microscopy images and 3D intensity plots of the focused light spots at different flow rates, i.e., 0 µL/min, 150 µL/min, 250 µL/min. This cylindrical lens based on Dean flow is still a 2D optical device. 

Through the effect of Dean flow and proper channel design, a series of 3D liquid waveguides and lenses have been demonstrated. Considering a 3D liquid waveguide or lens, the core flow stream was wrapped by the cladding flows. The transverse flow motion generated in the curved microchannel is the main cause for coating the core flow. There are two main factors that count for the realization of the 3D liquid core-liquid cladding structure. First, a relatively high Dean number is necessary to provide a large inertial centrifugal force. From the definition of the Dean number, it is feasible to increase the *De* by increasing the width of the channel (*w*) or decreasing the radius of curvature of the curved channel (*R*) or increasing the Reynolds number (*Re*) of the flow streams. Second, a long enough flow path is also an essential condition, which provides enough time for the transverse movement of the Dean flow under the effect of the centrifugal force. Another design recently reported by Yang et al., is a tunable dye laser based on a 3D optofluidic waveguide formed by centrifugal Dean flow, as shown in Figure 2c,d. In this optofluidic dye laser, a 3D liquid waveguide formed by two Dean flows acts as the gain medium. The core flow of the 3D waveguide was filled with laser dye. To fulfill the two main factors for the realization of the 3D liquid waveguide, the curved microchannel for Dean flow was designed with a substantially large curvature of radius (*R* = 2 mm) across 180 degrees. Once the microchannel was fabricated, the flow rates of the inlet flow streams would determine the formation of the 3D liquid waveguide. If the flow rates are relatively low, the inertia role of the fluids will not make a big difference. The inner flow is not completely wrapped by the outer flow. If the flow rates are too high, the position of the two flows will exchange. A proper 3D waveguide will not generate. The effect of centrifugal forces in a microchannel at different flow rates was shown in Figure 2d. Compared with traditional 2D optofluidic dye lasers, this 3D tunable dye laser possessed a higher energy output and a lower lasing threshold. The output energy of this 3D optofluidic dye laser can be varied by changing the flow rates of the two Dean flows in real-time. This liquid laser has the potential to provide a versatile tool for biosensors in optofluidic systems. Li et al., also designed a switchable 3D optofluidic Y-branch waveguide via Dean flows [51], as shown in Figure 2e. Two symmetrical curved microchannels with multi-curvature of radius, small (*R* = 1.5 mm, *arc* = 180°) and large (*R* = 2.5 mm, *ace* = 265°), and one Y-junction can be designed. Two independent 3D liquid waveguides can be formed through the separate curved microchannels. When coming across at the Y-junction, these two individual 3D liquid waveguides will combine into one main waveguide, Figure 2f. This Y-branch waveguide can guide light and realize switching the input light by adjusting the state of the independent 3D liquid waveguides, which are under the control of the flow rates of each side. The optical and transmission characteristics of the main 3D liquid waveguide are identical with multimode fiber. This device possesses large tunability and reconfigurability. The relative intensity of the output light at each branch can be adjusted from 1 to 0. When transmitting light with a wavelength of 532 nm in a situation of a 1:1 output ratio, the transmission loss of this 3D optofluidic Y-branch waveguide was estimated to be 0.97 dB. And the light spots at the output remained at a low level of deformation.

**Figure 3:**
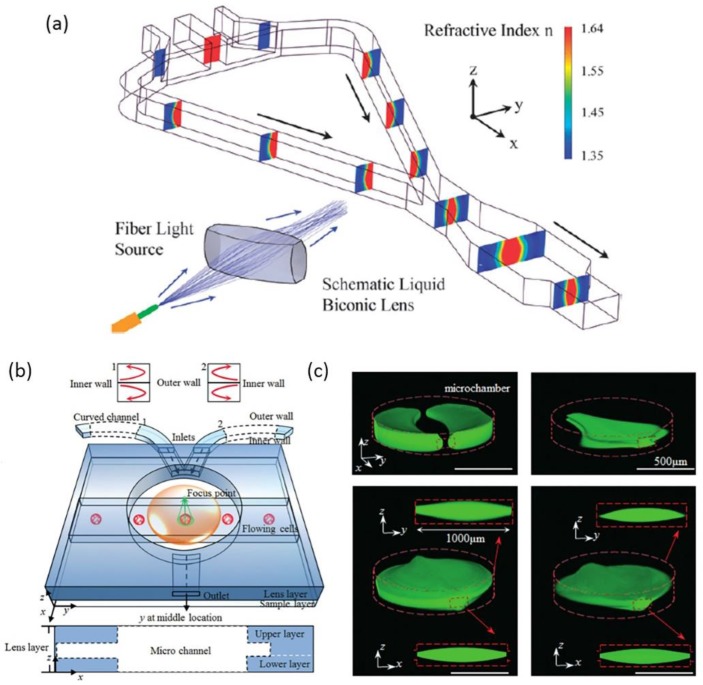
Schematic of the 3D fluidic lens (**a**); (**b**) Schematic diagram of the switchable 3D liquid–liquid lens for cell images; (**c**) The formation of the 3D lens under different flow rates. Images reproduced with permission from [50,52].

Aside from 3D liquid waveguides, Dean flow also allows for the design of 3D liquid lens. Rosenauer et al., presented the first tunable liquid biconic lens with the ability to focus light three-dimensionally [50], shown in Figure 3a. The light focusing in the direction of the X-axis was realized through the fabrication of an expanded microchannel, which shared the same method as in Figure 1c,d. In the direction of the Z-axis, the liquid interfaces of the 3D biconic lens were generated by inserting two curves (90 degrees). The combination of the vertical lens based on Dean flow and the horizontal lens based on the asymmetrical expanded chamber provides a high level of lens tunability. The design with separating functional parts also allows non-correlational modulation of the lateral and transversal curvatures of the lens. An ingenious design of a three-dimensional liquid biconvex lens combined by two symmetrical curved microchannels and an expanded circle chamber was demonstrated by Liang et al., to detect the living cells in flow streams [52], as shown in Figure 3b. Through the auxiliary curved microchannels, a structure of 3D-focusing of the core flow was formed based on Dean flow. As the 3D focused core stream flows into the expanded chamber, it widened in the horizontal and vertical directions and became biconvex in shape. Figure 3c shows the formation of the 3D lens under different flow rates. A wide range of variable focal lengths from 3554 µm to 3989 µm was achieved by changing the flow rates. The 3D liquid lens possesses a large numerical aperture ranging from 0.175 to 0.198, thus providing a higher imaging definition over a traditional objective lens. The images of two living cells, sp2/0 and NB4, were captured through the 3D liquid lens. This kind of 3D biconvex lens has the potential for application of real-time cell imaging and analysis in optofluidic systems.

## 4. Manipulation of the Gradient Refractive Index

An inhomogeneous liquid flow in optofluidic devices can be realized not only by controlling the liquid-liquid interface, but also by manipulating the gradient refractive index (GRIN). The GRIN in a microchannel can manipulate light propagating both perpendicular and parallel to the flow direction. Alternative approaches of creating GRIN in optofluidic devices are to form a concentration gradient or a temperature gradient. 

### 4.1. Liquid Diffusion and Transformation Optics

Miscible liquids and their interdiffusion can be of significant use in designing optofluidic devices. The diffusion process of two liquids is a unique characteristic that cannot be found in solid-based devices [53,54]. More concretely, liquid diffusion can create a concentration gradient, thus, forming a refractive index gradient. Here, diffusion becomes an advantage instead of a drawback. The distribution of the GRIN profile in the fluidic systems can be adjusted trough changing the flow parameters, such as *Pe*, or replacing different types of liquids that possess different refractive index and diffusion coefficients. The modulation of GRIN provides great flexibility and tunability to light manipulation for optolfuidic systems. For example, an optical splitter based on the merging of two parallel liquid waveguides has been demonstrated [53]. The diffusion, the refractive index, and the coupling degree between two separated waveguides were determined by the rates of flows. When the flow rate is slow enough to allow full diffusion of the two core flow streams in a microchannel, the two parallel liquid waveguides will smoothly merge into a single waveguide. The input beam will be split into two output beams with an intensity ratio of 1:1 when light propagates in the opposite direction of the flow streams. Unlike a conventional beam splitter, the split ratio of the optofluidic beams splitter can be dynamically tuned. Changing the flow rate changes the gradient of the refractive index, and thus the output ratio and intensity of the liquid beam splitter.

**Figure 4:**
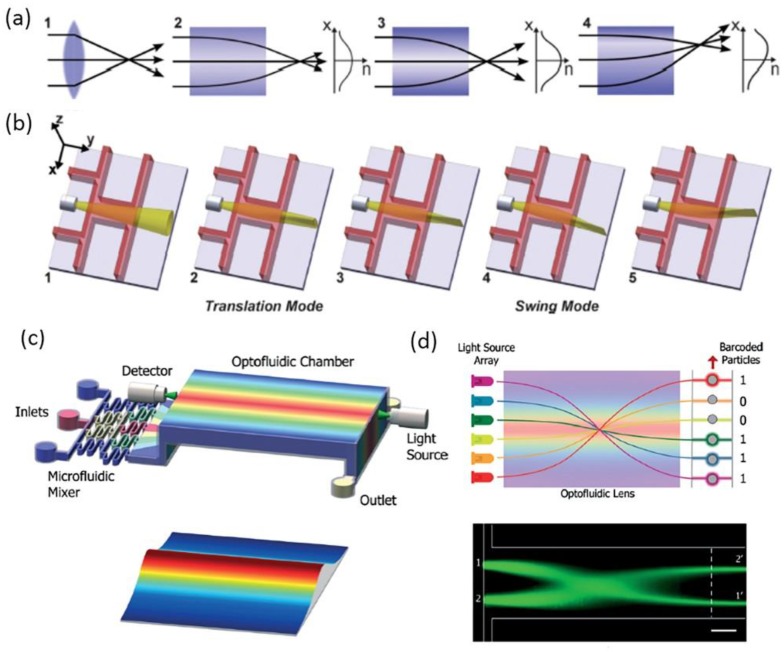
Principle and design of the liquid gradient refractive index L-GRIN lens (**a**,**b**); (**c**) Schematic illustration of the optofluidic lens with low spherical and low field curvature aberrations and its Stable RI distribution; (**d**) Schematic illustration of the potential application in multiplexed detection. Images reproduced with permission from [54,55].

For a typical straight liquid waveguide consisting of three laminar flows, the refractive index profile in a microchannel can be illustrated by Equation (8). Through the manipulation of flow streams, a GRIN distribution can be formed to guide the flow of light. Mao et al., reported a tunable liquid gradient refractive index optofluidic lens for light focusing [54]. Instead of using the step-index interface between curved fluids, a GRIN across the liquid medium was applied to focus and bend light beams, as shown Figure 4a. A hyperbolic secant RI profile, which was suitable for light focusing, was established through the diffusion of CaCl_2_ solute between the sandwiched core flow (CaCl_2_ solution) and cladding flows (DI water). Both the focal length and the output direction of light can be tuned by changing the flow rates, the two corresponding working states were shown in Figure 4b. This design of GRIN microlenses has two degrees of freedom, which provides large flexibility and novel functionality for light manipulation. In a later design, Liu et al., reports an optofluidic lens with low spherical and low field curvature aberrations, as shown in Figure 4c [55]. A hyperbolic secant (HS) refractive index profile was generated by adjusting the diffusion between ethylene glycol and deionized water. The spherical aberration in this optofluidic lens HS profile is much lower than that in the former design of liquid GRIN lenses. Owing to the small spherical aberration, the optofluidic lenses have found applications in the manipulation of light source array and multiplexed detection, as shown in Figure 4d.

**Figure 5:**
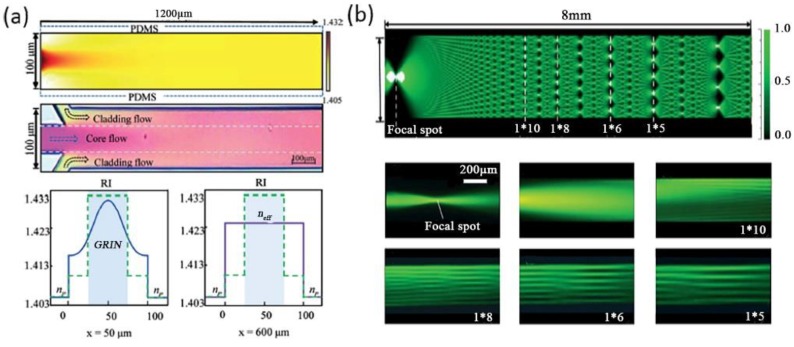
Self-imaging effect by MMI in the hybrid optofluidic waveguide. (**a**) Stable RI distribution in the main channel; (**b**) The self-imaging interference pattern. Images reproduced with permission from [56].

Aside from light focusing and bending, the GRIN of liquid flows also allows for light interference and diffraction. Shi et al., reported a tunable multimode interference (MMI) device as shown in Figure 5a,b [56]. The MMI device consists of two main modules: a GRIN liquid lens and a step-index liquid/solid interface. Three liquid flows with relatively low flow rates were injected to form a gradient RI, thus focusing light and realizing the modulation of MMI. Owing to the chosen low flow rate, full diffusion mixing was realized in the first part of the hybrid waveguide. In the latter part of the optofluidic waveguide, the liquid solution became homogenous so as to form a step-index distribution with the wall of the microchannel. The RI of the core and cladding flows were chosen at 1.432 and 1.410, which were both higher than that of the microchannel (1.405), to obtain a relatively low optical loss of the liquid system. The refractive index profile suited for MMI of the hybrid waveguide was shown in Figure 5a. Figure 5b shows the light focusing and interference patterns at different positions, which was in good agreement with the simulation result. In addition, the period of MMI can be modulated by simply adjusting the flow rates or RI.

Transformation optics is a fantastic kind of mathematical technique that provides a means to design complex artificial media using the invariance of Maxwell equations in certain coordinate transformations [57]. The artificial media with spatially changing permeability and permittivity offers precious control of the flow of electromagnetic waves. The most striking example is the “invisibility cloak” demonstrated by Pendry et al., in 2006. With the advancement of metamaterials, a wide range of optical devices have been realized through the method of transformation optics, including beam shifters, bent waveguides, beam splitters, Luneberg lenses, dielectric cloaks, and carpet cloaks [58,59,60,61,62,63]. However, the realization of artificial metameterials suffers from complex design and fabrication processes. It is difficult to construct transformation optical devices with large object size by using metameterials. The operating wavelengths of these solid transformation optical devices are limited by the feature sizes of their nano-structures. It is difficult to operate at the visible light band. In optofluidic systems, the GRIN liquid media formed by diffusion between miscible flows at low-Pe level has the potential to be a new kind of material system. And it provides a versatile tool for designing transformation optical devices with controllable and spatially changing optical properties.

**Figure 6:**
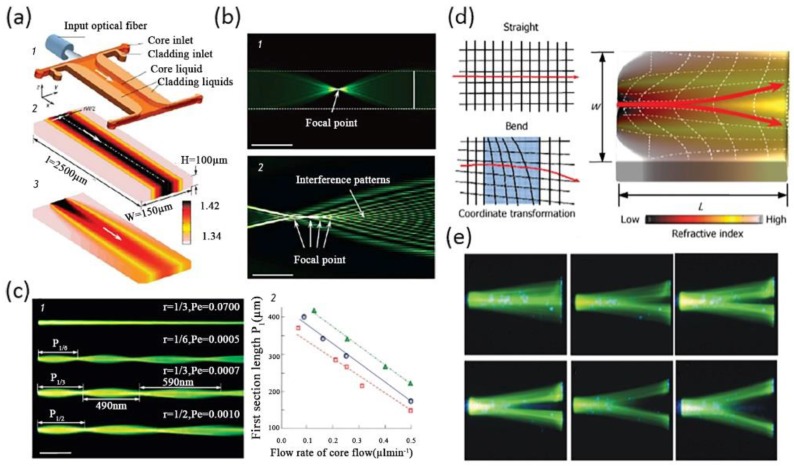
(**a**) Design of the optofluidic waveguides via a transformation optics approach; (**b**) Light focusing and interference in an optofluidic waveguide. The light trajectory and converging points as a function of the Pe and the flow rate (**c**); (**d**) Schematic illustration of the pure liquid optofluidic Y-branch splitter; (**e**) A wide range of split angle, from 0° to 30°, can be achieved by choosing the proper flow rates. Images reproduced with permission from [64,65].

Based on the concepts above, researchers have used liquid flows as a new tunable transformation optics (TO) medium in optofluidic devices to manipulate light. Various fancy optofluidic TO devices were designed with novel optical properties. One of the most representative and fundamental designs was the liquid TO waveguide [64,65], and examples are shown in Figure 6. Compared with conventional liquid waveguides with high flow rates or high *Pe*, the optofluidic TO waveguide made full use of liquid diffusion between three flow streams to generate an inhomogeneous GRIN field in the transverse direction and the bidirectional direction, shown in Figure 6a. By changing the flow rates in a single liquid waveguide, spatially variable optical properties will be created to support novel optical phenomena such as self-focusing and interference [64], as shown in Figure 6b. The interference pattern in this optofluidic TO waveguide was similar to that in discrete diffraction. In addition, traditional discrete diffraction is usually generated in solid waveguide arrays whose feature sizes were several micrometers. It is fantastic that one can create a typical interference pattern through a simple liquid waveguide rather than solid waveguide arrays fabricated by complex processes. Furthermore, this liquid waveguide also possessed high tunability. As shown in Figure 6c, the light trajectory and converging points differed as the *Pe* decreased from 0.0100 to 0.005. The focusing period also increased because of a GRIN along with the direction of flow stream. In a relatively high *Pe* (0.07), the light traveled by straight lines. The relationship between the first section lengths and the flow rate of the core fluid in different boundary ratio was also shown in Figure 6c. In a later design, Yang et al., introduced a transformation Y-branch splitter by using an ethylene glycol solution as the high *RI* (*n* = 1.432) cladding flows and deionized water as the low *RI* (*n* = 1.333) core flow [65]. The RI-profile in this Y-branch splitter is bi-directional, as shown in Figure 6d. The flow rates of each input channel were calculated referring to the coordinate transformation for accurate light splitting. As a result, a wide range of split angles, from 0° to 30°, was achieved by choosing the proper flow rates, as shown in Figure 6e.

**Figure 7:**
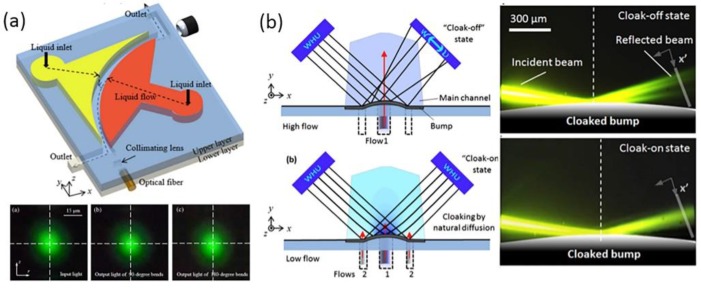
(**a**) Main concept of liquid waveguide bends and the light beam profiles of the liquid bends; (**b**) The “cloak-off” and “cloak-on” state of the switchable optofluidic carpet cloak using miscible liquids. Images reproduced with permission from [66,67].

It is unique that the convection–diffusion equation at low *Pe* shares the same mathematical form with quasi-conformal transformation optics (QCTO). Based on the concept of transformation optics not only have transformation optofluidic Y-branch splitters and waveguides been demonstrated, various devices like tunable waveguide bends and tunable liquid visible cloaking devices have also been designed for light manipulation by controlling the diffusion of liquid [66,67]. Figure 7a shows a tunable liquid TO waveguide bend, developed by Liu et al., The bend was formed by choosing the special boundary conditions of the diffusion process between ethylene glycol and deionized water. A gradient refractive index profile that coincided with that of the TO bend waveguide was achieved, thus steering the light path without optical loss in the same way as other TO systems [66]. The light beam profiles at the input and the output of the 90° bend and the 180° bend were almost the same, which means that the light beam maintained perfectly through the TO liquid waveguide. The manner of light in GRIN liquid waveguide bends was the same as that in homogenous straight liquid waveguides. Zhu et al., also creatively designed a tunable visible cloak by liquid diffusion [67], as shown in Figure 7b. A bump was designed at the bottom of the main channel to hide objects. It was easy to change the working states of this liquid visible cloak by controlling the motion of the three inlet miscible flows. When the RI profile in the main channel mismatched with that of the QCTO, the incoming light is scattered by the bump. The liquid visible cloak was in a “cloak-off” state. As a result, the object behind the bump could be detected. In contrast, if the miscible flows were injected at enough low flow rates, an inhomogeneous *RI* profile showing analogy with that of QCTO generates. The liquid cloak maintained a “cloak- on” state. The reflected light ray in this device was the same as that in a flat mirror so that objects inside the bump were hidden.

### 4.2. Heat Conduction

The refractive index of a liquid is almost always related to its temperature. The temperature distribution through the heat conduction or thermal diffusion between several fluids in a microchannel with different temperatures will create a gradient refractive index profile similar to mass diffusion. This GRIN profile of the liquids in return will guide the propagation of the light beam [68]. Compared with traditional mass diffusion, the thermal diffusion can form an inhomogeneous GRIN profile in a homogeneous liquid flow. The use of a homogeneous liquid flow or single common liquid may simplify the liquid recycling process. In addition, thermal diffusion is more rapid than mess diffusion, which offers an opportunity for reducing the responding time of the optofluidic system. But these optofluidic devices based on heat conduction suffer from a relatively low *R*I difference, which limits the capability of manipulating light beams. Besides, an extra thermal field is needed to maintain the sustained inputs of liquids with a specific temperature different from room temperature. 

The temperature distribution across the channel is controlled by the heat conduction equation:

(9) $$\frac{\partial T}{\partial t}=\psi \nabla^{2}T-U\nabla T$$

where *T* is the temperature, U is the average velocity of the liquid in microchannel. $\psi =k/\rho C_{p}$ is the thermal diffusivity, *k* is the thermal conductivity, ρ is the density of liquid, and $C_{p}$ is the specific heat. The adjusting of RI can be realized by changing the temperature. According to the thermo-optics effect, the relationship between RI of a liquid and the temperature is expressed by

(10) $$n\left(T\right)=n_{0}+\varepsilon \left(T-T_{0}\right)$$

where $n_{0}$ is the RI at the initial temperature , and ε is the thermal coefficient of the liquid.

**Figure 8:**
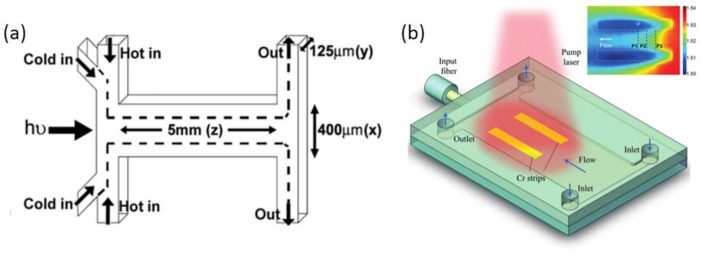
(**a**) Schematic design of the liquid thermal GRIN optical waveguides; (**b**) Schematic diagram of the optofluidic tunable lenses using laser-induced thermal gradient. Images reproduced with permission from [68,69].

Tang et al., describes the design of a liquid thermal optical waveguide [65], as shown in Figure 8a. The refractive index of a liquid usually keeps negative correlation with the temperature. A flow stream with a lower temperature (21 °C) was sandwiched by two flow streams with a higher temperature (range from 30 °C to 80 °C). The heat conduction between the core flow and cladding flows results in a gradient RI distribution across the microchannel. This design enables the control of heat diffusion and then the RI by controlling the initial temperature difference and the flow rates in each input channel. In a later design, an optofluidic lens based on a laser-induced thermal gradient was demonstrated by Zhang et al., as shown in Figure 8b [69]. Compared with Tang’s method, this approach was realized by an extra optical field instead of inserting liquids with different temperatures. Two straight chromium strips were fabricated at the bottom of the channel to absorb the energy of a pump laser. In this liquid thermal lens, benzyl alcohol solution was used because a relatively larger refractive index change can be obtained compared with other liquids such as water under a certain temperature difference. A 2D refractive index gradient will be formed between the two hot strips. It is demonstrated that the focal length can be continuously tuned from infinite to 1.3 mm. At the same time, an off-axis focusing can be realized by offsetting the heat spot of pump laser. This tunable lens possesses many advantages, such as small size, easy integration, and fast responding speed. However, it requires a more complex fabrication process and an extra optical field than previous thermal lenses. The efficiency of the laser-induced heating process is relatively low. Liu et al., also reported a liquid thermal GRIN lens in homogeneous fluids [70]. The focal length of the thermal lens can be adjusted from 500 μm to 430 μm. In this design, a relatively high enhancement factor can be achieved (5.4). And the corresponding full width at half maximum was 4 μm.

## 5. Application in Biochemical Sensing

The capabilities of light manipulation and biochemical sensing are inherent along with the emergence of the optofluidics. The liquids in optofluidic systems are natural carriers for biological samples (i.e., cells, prokaryotes, DNA), nanoparticles, molecules like phosphate, and other water-soluble components. Optofluidics has the advantage of being highly-integrated, low-cost, fast in the field of biochemical sensing, detection and particle manipulation [12,13,15,16]. Optofluidics makes full use of the powerful tools and techniques in optics, such as evanescent wave fields, optical tweezers, and resonant cavity to enhance the function and efficiency of traditional microfluidic systems [71,72,73,74,75]. 

**Figure 9:**
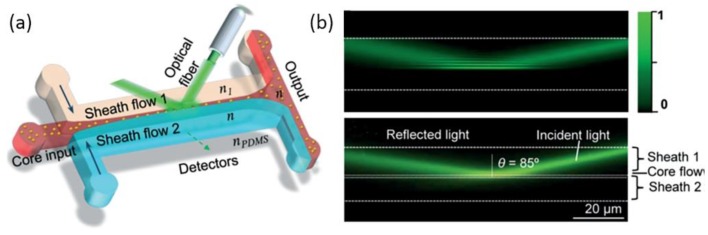
(**a**) Design of the optofluidic chip for single nanoparticle detection; (**b**) Optical intensity distribution of the evanescent field. Images reproduced with permission from [76].

An evanescent wave field is one of the most widely used technologies in the detection of single nanoparticles/molecules with high sensitivity and signal-to-noise ratio. However, only a few samples in liquid can be illustrated by the evanescent wave at the solid-liquid interface. The samples are detected randomly. In order to overcome these drawbacks, one can either increase the intensity and the penetration depth of the evanescent field or confine the samples within the area illustrated by the evanescent wave. On the basis of these two approaches, Liang et al. demonstrated an optofluidic chip for nanoparticle detection [76], shown in Figure 9a. A silicone oil and paraffin oil mixture with high-RI was used as the sheath flow. While the low-RI ethylene glycol solution was used as the core flow. The interaction between the two immiscible fluid flows will generate an optically smooth and step-index interface, which was suitable for total internal reflection. By choosing the proper parameter of the RI and incident angle, the penetration depth can be broadened up to 1 µm as shown in Figure 9b. The sample core flow was then focused with a width narrower than 1 µm through the method of hydrodynamic focusing. This optofluidic chip realized the detection of every sample in the core flow through an evanescent wave without any failure. The application of TIR into optofludic systems will promote the improvement of detection systems with real-time control and rapid responses.

**Figure 10:**
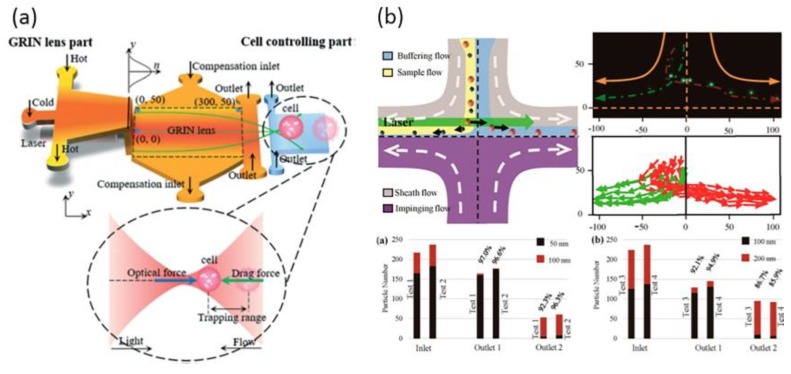
(**a**) The design of the liquid thermal GRIN lens for trapping living cells in a dynamic liquid environment; (**b**) Precise sorting of gold nanoparticles in a flowing system where the sorting efficiency is as high as 92%. Images reproduced with permission from [70,82].

Particle manipulation and sorting is another main application of optofluidcs. The optical tweezer is an efficient and effective tool for trapping micro particles by applying a strong focused laser beam [77]. Optical tweezers, especially holographic optical tweezers (HOTs) have been applied in the fields of biology, optical manipulation, and channel-facilitated diffusion [78,79,80]. By combining the microfluidic array and HOTs creatively, researchers have succeeded in investigating single-file diffusion of Brownian particles [81]. A solid objective lens is usually applied to form optical tweezers in conventional approaches. As shown in Figure 10a, based on the unique optical properties of the thermal GRIN lens, Liu et al., realized the trapping of a single living cell in a dynamic liquid environment [70]. By varying the focus length of the optofluidic device, the living cell can be trapped at different locations, which provides an approach for the manipulation and analysis of a single living cell. Wu et al., combined the optical force and the opposite impinging streams to achieve the size-selective optical sorting of gold nanoparticles in fluids [82], as shown in Figure 10b. The injection of the two opposite laminar streams meeting at the junction generate a smooth stagnation point, which will decelerate the moving nanoparticles. In other words, this design of the impinging streams can prolong the function time of optical force. In return, this optofluidic sorter for NPs owns higher efficiency than other sorting methods, such as centrifugation, electrophoresis, and size exclusion etc. In the experiment, the sorting of different-sized nanoparticles is demonstrated successfully. The sorting efficiency for 50/100 nm and 100/200 nm mixtures are 92% and 86%, respectively. A sorting output of 300 particles per minute was realized. 

**Figure 11:**
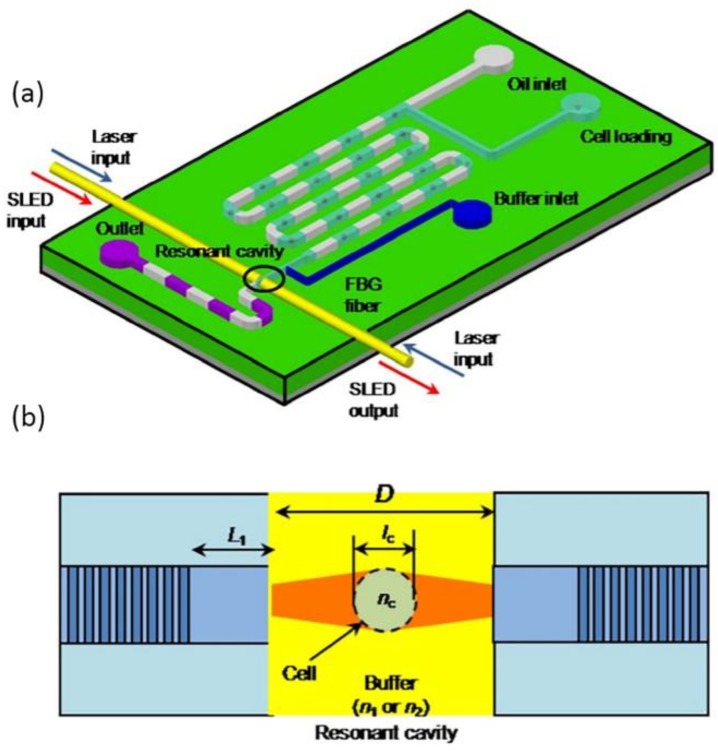
(**a**) Schematic diagram of the biochip design and (**b**) the fiber Bragg grating resonant cavity. Images reproduced with permission from [85].

In addition, several optofluidic chips were demonstrated to measure the effective refractive index of living cells through novel optical techniques such as Fabry–Pérot (FP) resonant cavity, fiber Bragg grating resonant cavity, and the Mach–Zehnder interferometer etc. [83,84,85]. The resolution of the refractive index unit (RIU) can reach the order of 10^−3^. A typical cell refractive index model is shown in Figure 11a,b. Aside from applications in particle and cell sensing and manipulation, optofluidics also finds important applications in environmental detection. By the combination of fluid control and optical detection in the scale of micron meters, optofluidic biochemical devices could be designed with small size, low cost, parallel processing, and real-time monitoring. Zhu et al., reported a lab-on-a-chip analysis system for phosphate detection [86]. A FP resonant cavity was fabricated by two opposite aligned Au-coated fibers to enhance the absorption of phosphate. Compared with traditional spectroscopy instruments, the optofluidic phosphate detector design possesses several superiorities such as miniaturization with short absorption cell length down to 300 µm and fast detection (6 s). 

## 6. Summary and Outlook 

This paper reviews optofluidic lab-on-a-chip techniques based on an inhomogeneous liquid flow for light manipulation and demonstrates some application examples in biochemical sensing. Optofluidic lab-on-chip manipulation techniques and designs are categorized according to the interaction between different flow streams. Emblematical and significant works are introduced from the perspectives of manipulation of a liquid-liquid interface and that of the liquid gradient refractive index. In these woks, researchers find ways to control the liquid in a microchannel, realizing light routing, bending, switching, focusing, and interference etc. Fluids can be easily reconfigured and replaced, allowing for much larger tunability in the refractive index and flexibility in shape than solid equivalents. By manipulating flow rate and liquid compositions, the function of the optofluidic light manipulation devices or systems can be fully exploited. Besides the tunability and reconfigurability, optofluidic devices possess another outstanding advantage, easy integration. Compared with conventional optical systems, optofluidic devices can be fabricated and integrated in other MEMS chips as an optical control element.

In the future, researchers may focus on new microfluidic liquid control methods and potential techniques to improve the performance of optofluidic systems. Recently, transformation optics has drawn a lot of intention. Researchers will come across new propositions and research points after applying the concepts and designing new methods of transformation optics in optofluidics. As a result, the marriage of transformation optics and optofludics will promote various novel qualities in light manipulation. No matter what the future will be, the optofluidic lab-on-a-chip system based on pure liquid is a powerful concept for light manipulation. Based on this, increasing optofluidic systems or devices for real-time monitoring with properties of fast, accurate, low-cost, small-sized biochemical micro- sensors will be brought into existence.

## Figures and Tables

**Table 1:** Comparison of optofludics with other light manipulation techniques in relative refractive index change (*D*) and responding time (*τ*) [1].

Technology	*σ* (Δn/n)	τ(s)
Optofluidics	1	10^−3^
Liquid crystal	10^−1^	10^−3^
Injection current	10^−2^	10^−9^
Temperature	10^−2^	1
Photorefractive	10^−3^	10^−1^–10^−5^
Electro-optic (10 kV/cm)	10^−3^	10^−12^
Photoelastic/Acousto-optic (10 W)	10^−4^	10^−6^–10^−7^
